# The cortisol awakening response at admission to hospital predicts depression severity after discharge in major depressive disorder patients—A replication study

**DOI:** 10.3389/fnins.2022.952903

**Published:** 2022-10-12

**Authors:** Sabrina Neyer, Michael Witthöft, Mark Cropley, Markus Pawelzik, Stefan Sütterlin, Ricardo G. Lugo

**Affiliations:** ^1^EOS-Klinik, Münster, Germany; ^2^Department for Clinical Psychology, Psychotherapy and Experimental Psychopathology, University of Mainz, Mainz, Germany; ^3^School of Psychology, University of Surrey, Guildford, United Kingdom; ^4^Faculty for Health and Welfare Sciences, Østfold University College, Østfold, Norway; ^5^Hochschule Albstadt-Sigmaringen, Sigmaringen, Germany

**Keywords:** cortisol awakening response, depression, HPA axis, biomarker, follow-up, replication study

## Abstract

The cortisol awakening response (CAR) is a non-invasive biomarker for hypothalamic-pituitary-adrenal axis (HPA) dysregulation, reflecting accumulated stress over time. In a previous study we reported that a blunted CAR before an inpatient treatment predicted self-reported depressive symptoms six weeks and six months after discharge [Eikeseth, F. F., Denninghaus, S., Cropley, M., Witthöft, M., Pawelzik, M., & Sütterlin, S. (2019). The cortisol awakening response at admission to hospital predicts depression severity after discharge in major depressive disorder (MDD) patients. Journal of Psychiatric Research, 111, 44-50)]. This replication study adopted an improved overall methodology with more stringent assessment protocols and monitoring. The longitudinal design included 122 inpatients from a psychosomatic hospital with a diagnosis of MDD displaying symptoms of moderate to severe major depression (*n* = 80 females). The CAR was measured at intake. Depression severity was assessed as Beck Depression Inventory II scores at intake, discharge, 6 weeks and 6 months following discharge. Results from the original study were replicated in terms of effect size but did not reach statistical significance (correlation between BDI-II 6 months after discharge and AUCg: *r* = −0.213; *p* = 0.054). The replication study yielded nearly identical correlation coefficients as in the original study (BDI-II 6 months and CAR, *r* = −0.223, *p* < 0.05). The replication of previously reported effect sizes with a concurrent lack of statistical significance in the more restrictive, larger and better controlled replication study may well inform research on psycho-endocrinological predictors for treatment success, but suggests a rather limited practical relevance for cortisol awakening response measures in this clinical context.

## Introduction

Major depressive disorder (MDD) affects 4.4% of the worldwide population with prevalence estimates rising steadily, yet there remains a lack of sufficiently effective and sustainable treatment options (World Health Organization [WHO], 2017). One possible explanation for this, is the heterogeneity of MDD and the associated heterogeneous diagnostic tools, treatment possibilities and different treatment results (Baumeister and Gordon, 2012; Van Loo et al., 2012). Research is therefore trying to find markers that may help to classify and specify symptoms to adapt to individual therapy. The extent to which treatment can become more effective for MDD arguably depends on a deeper understanding of its etiology and pathophysiology (Saveanu and Nemeroff, 2012).

Valid biological markers which may help to specify the diagnosis and predict post treatment symptom deterioration are still scarce. This may be due to the large heterogeneity of physical symptoms associated with MDD (e.g., sleeping disorder, reduced appetite, pain, blood count changes, and many more), the insufficient measurement validity of many biomarkers (Mayeux, 2004), or the lack of measurement guidelines.

### Biological foundations

Some studies report that MDD patients have typical alterations in the hypothalamic-pituitary-adrenal (HPA) axis (Ehlert et al., 2001; Varghese and Brown, 2001). Most MDD patients demonstrate hypersecretion of cortisol partly due to an impaired endogenous glucocorticoid feedback regulation of HPA axis activity (Stetler and Miller, 2011; Saveanu and Nemeroff, 2012). Cortisol secretion in the after awakening is considered to be one of the most relevant measures to classify the function of the HPA axis (Stalder et al., 2016). In response to awakening, cortisol secretion rises sharply and peaks after approximately 30–45 min; a process termed the “Cortisol awakening response” (CAR) (Pruessner et al., 1997; Fries et al., 2009). The literature points out that CAR has been related to the anticipations for the upcoming day and “mobilizing” of energy resources (Adam et al., 2006; Fries et al., 2009). The CAR represents a reliable measure of the HPA axis reactivity, that is sensitive to different psychosocial and health factors such as job stress, life stress and fatigue (Schmidt-Reinwald et al., 1999; Chida and Steptoe, 2009). Chronic and acute psychosocial stress has been associated with an increased CAR in cross-sectional studies (Schlotz et al., 2004; Adam et al., 2006).

### Cortisol awakening response in major depressive disorder patients

Studies examining the association between the CAR and depression have so far found that an unusual (higher or lower) CAR is significantly associated with depression; but the direction of this association seems unclear. Some studies report a reduced CAR in depressed patients (Stetler and Miller, 2011; Dedovic and Ngiam, 2015; Adams et al., 2020), whereas others report an increased CAR (Pruessner et al., 2003; Bhagwagar et al., 2005). Recent research, however, suggests that the CAR association is moderated by depression severity, in which mild to moderate degrees of depression have been related to a heightened CAR, while severe or chronic depression to a blunted CAR (Chida and Steptoe, 2009; Wardenaar et al., 2011). A long period of mental or physical stress leads to a downregulation of cortisol receptors so that the HPA axis becomes less responsive (Heim et al., 2000). Fries et al. (2005) suggest that hypocortisolism results from a long period of hypercortisolism.

To date, there is a lack of clarity and debate as to whether a changed CAR is a risk factor for the development of depressive episodes or a consequence of the disorder. There is evidence from several prospective studies that a high CAR might be a biomarker in healthy young people to predict the onset of MDD (e.g., Mannie et al., 2007; Adam et al., 2010). But there is also research suggesting that a changed CAR might be a consequence of the disorder and also a predictor of the course of the disorder (Bhagwagar and Cowen, 2007; Vreeburg et al., 2013). For example, Bhagwagar and Cowen (2007) found elevated cortisol secretion in recovered depressed patients, while Vreeburg et al. (2013) found that a lower CAR in depressive patients, predicted an unfavorable course of disorder development over the following two years.

### Changes of cortisol awakening response after psychotherapy

Jones et al. (2015) argue that the CAR might be a predictor for treatment outcomes in MDD patients. Patients with a high CAR at intake showed a better treatment response at discharge after a 4-week hospital stay. At follow-up measurement points, however, many MDD inpatients show a typical symptom deterioration which may be influenced by the renewed confrontation with pretreatment stressors in their environment (Monroe et al., 2009). Possible explanations for these findings could be that depressed individuals may elicit social rejection through maladaptive social behavior (Van Orden and Joiner, 2013). Refsgaard et al. (2022) show that higher CAR is associated with less improvement of depression inpatient psychiatric therapy.

To further investigate these results, our earlier study (Eikeseth et al., 2019) analyzed the association between CAR at intake and long term depressive symptoms in a naturalistic inpatient clinical setting. We were able to show that a blunted CAR before psychotherapy is related to the severity of depression six weeks and six months after discharge in patients with MDD. However, the effect sizes were small, and as the observation period of 30 min after waking up was used, it remains questionable whether the true CAR peak was measured. Additionally the review from Bhagwagar and Cowen (2007) shows that many of the neurobiological abnormalities still persist after a depressive episode and that these abnormalities are associated with changes in emotional information processing. They show that in people where the risk of recurrent depression is increased, the brain still appears to be in a state that prefers the processing of negative information.

### Need of replication study

In summary, the CAR might be a possible candidate to provide some information on the course of MDD at the beginning of a psychotherapy, but so far there is a lack of studies that have examined the long term effects, particularly in naturalistic settings. There are concerns about the replicability of the relationship between CAR and depression and therefore there is a call for replication studies to get more information about the association and predictive power of CAR (De Weerd-Wilson and Gunn, 2017). Therefore the purpose of this study is to analyze the CAR during the first days of hospitalization prior to a naturalistic inpatient psychotherapeutic treatment to predict long-term treatment outcome in MDD. We do this by replicating our previous study from Eikeseth et al. (2019) following a now stricter measurement and monitoring protocol (CAR measurement and monitoring protocol as suggested by Stalder et al., 2016). While Eikeseth et al. (2019) measured the CAR two times in the morning (awakening and 30 min after awakening), this assessment protocol could be criticized for potentially missing the CAR preak, even though that at least 50% mean cortisol increase occurs within the first 30 min after awakening (Pruessner et al., 1997; Wüst et al., 2000). The replicating study uses a longer measurement interval and three samples to determine a more robust way of calculating CAR as area under the curve to predict depressive symptoms 6 weeks and 6 months after discharge. In a written follow-up survey after the cortisol measurement, compliance with the measurement protocol, sleep, mood and wake-up times were ascertained.

### Hypotheses

Based on our previously reported findings (Eikeseth et al., 2019), we hypothesized that the CAR at intake would be significantly negatively associated with follow-up depressive symptoms and predict symptom deterioration 6 weeks and 6 months following an inpatient psychotherapy.

## Materials and methods

### Participants

The sample of this study is highly comparable to Eikeseth et al. (2019). One-hundred and forty nine inpatients (67.8% females) admitted for psychotherapy treatment in a German psychosomatic hospital were recruited between 2019 and December 2020. As in the study of Eikeseth et al. (2019) the inclusion criteria was MDD as a main diagnosis. Exclusion criteria were glucocorticoid medication use, comorbid addiction disorder, excessive substance abuse, psychosis, autoimmune-thyroiditis, personality disorders due to medical conditions, respiratory disease, hormone or heart conditions (*N* = 21). The participants’ diagnoses were determined through a structured clinical interview (SCID I, II; Wittchen et al., 1997a,b) by a trained psychotherapist. Eighty-five percent (*N* = 127) of the patients suffered from at least one additional mental disorder. Therefore, most patients were on at least one psychotropic drug at intake or started a medication during the psychotherapy. This study was approved by the Ethics committee of the “Medical Association Westfalen-Lippe” and written informed consent was obtained from all participants prior to data collection. This study was also pre-registered on Aspredicted.org (#45146).

### Materials and procedures

#### Beck Depression Inventory-II

Consistent with Eikeseth et al. (2019) we used the Beck Depression Inventory-II (BDI-II; Beck et al., 1996a). This is a self-assessment questionnaire which indicates the intensity of depressive symptoms and attitudes in accordance with the DSM-IV criteria (Beck et al., 1996b; German version: Kühner et al., 2007). All assessments were completed via an online questionnaire. The patients were personally contacted by a member of the research/clinical team by email six weeks (BDI-II 6WF) and six months (BDI-II 6MF) after discharge. After completing each follow-up assessment, the participants were invited back to the clinic to discuss their results with their individual psychotherapist.

#### Assessment of cortisol awakening response

As in the study by Eikeseth et al. (2019), the cortisol secretion was assessed during the first 5 days of admission through saliva sampling using cotton salivettes (manufacturer: Sarstedt AG & Co., Nümbrecht, Germany). In this study we assessed the cortisol level at three time points in the morning (Eikeseth et al., 2019), which were directly after awakening in the morning, 30 min after awakening, and 45 min after awakening. The samples were sent to a medical laboratory for Enzyme Immunoassay after collection on the same day. The CAR was calculated by using the Area Under the Curve ground (AUCg) and the Area Under the Curve increase (AUCi) in line with Pruessner et al. (2003). Persons with an AUCg of at least.091 μgdl were classified as CAR responder (Clow et al., 2004). The AUCg has been shown to be a reliable marker in terms of individual stability (Edwards et al., 2001). Additionally, we calculated the total cortisol increase, calculated as the difference between cortisol levels at 30or 45 min after awakening and cortisol right after awakening. The calculation of the more accurate and widely accepted AUC measure in addition to cortisol increases assessed as difference between two time points is an extension of the original investigation by Eikeseth et al. (2019).

Salivettes were handed out by the therapists the day before the measurements along with instructions about using the first salivette directly after awakening, the second salivette 30 min after awakening and the last one 45 min after awakening. Participants were advised to refrain from brushing their teeth, sucking on drops, doing exhausting exercise, caffeine consumption and smoking, between awakening and the following cortisol assessments after awakening.

In this study we added a follow up measure to ensure that the patients had fully and correctly implemented the instructions. Apart from that the patients had to indicate deviations from the survey protocol. In the event of serious deviations from the survey protocol, the measurement was repeated the following day or the patients were excluded from the study (*N* = 6). Participants were asked to specify at what time in the morning they woke up and if they had any sleeping disorders during the night.

After checking all exclusion criteria *N* = 122 patients were included into the study (*N* = 80 females; *N* = 42 males; Age = 39.33 years, SD = 14.35).

### Statistical analyses

Statistical analysis was performed using IBM SPSS Statistics version 27. Prior to analysis, all variables were checked for accuracy of data entry and missing values. The differences in sample size within the tables reflect missing values. Little’s MCAR (missing completely at random) Test showed a statistically non-significant result (χ^2^ = 42.54, *df* = 48, *p* = 0.696) indicating that values missing completely at random could be inferred (Tabachnick and Fidell, 2013). All variables were checked for univariate outliers by identifying cases with *z-*values above 3.29 or below −3.29, and dealt with by deletion. One individual’s measurement at the evening was identified as outlier and deleted.

The associations between CAR values and the BDI-II measurements (BDI-II Intake, BDI-II Discharge, BDI-II 6WF, and BDI-II 6MF) were assessed using Pearson’s correlations (significance level *p* = 0.05).^1^

## Results

### Descriptive statistics and check for potential confounding variables

We analyze the correlation between cortisol values at the beginning of an inpatient psychotherapy and depressive symptoms after inpatient psychotherapy. The descriptive statistics are presented in Table 1 including the CAR values. CAR values were lower compared to studies with healthy participants (see Clow et al., 2004, Cortisol Awakening = 0.40, Awakening + 30 = 0.73, Awakening + 45 = 0.67) and lower than in the Study from Eikeseth et al. (2019).

**TABLE 1:** Summary of descriptive statistics and mean cortisol levels.

		Replication study					Eikeseth et al., 2019 (Cortisol values transformed in μ g/dl)				
			
		*N*	*M*	*SD*	*Min*	*Max*	*N*	*M*	*SD*	*Min*	*Max*
Survey and Demographics	Age	118	39.34	14.35	18	67	128	43.1	13.7	17.0	68.0
	BDI-II Intake	122	31.20	10.19	14	56	127	28.7	11.8	1.0	52.0
	BDI-II Discharge	122	16.80	11.69	0	51	123	13.2	10.6	0.0	47.0
	BDI-II 6WF	120	17.50	12.60	0	52	93	15.0	11.8	0.0	49.0
	BDI-II 6MF	86	19.86	15.25	1	61	92	15.6	12.5	0.0	53.0
	Valid N						62				
Biomarker	Cortisol awakening	117	0.29	0.18	0.04	0.82	118	0.97	0.52	0.11	2.94
	Cortisol + 30	118	0.49	0.28	0.04	1.39	118	1.51	0.72	0.12	3.51
	Cortisol + 45	119	0.48	0.28	0.04	1.26					
	CAR + 30	117	0.20	0.21	−0.23	0.91	116	0.53	0.61	−0.71	2.23
	CAR + 45	117	0.19	0.24	−0.36	0.89					
	AUCi	117	5.91	6.37	−7.87	27.15					
	AUCg	117	18.80	10.26	1.79	47.02					
	N	117					112				

Cortisol Values in μg/dl. BDI-II, Beck Depressive Inventory II; WF, week Follow-up; MF, month follow-up; CAR, cortisol awakening response; AUCi, area under the curve increase; AUCg, area under the curve ground; M, mean; SD, standard deviation; Min, minimum; Max, maximum.

The examination of potential confounding variables with the primary key variables of CAR (AUCi and AUCg) and the depression severity at the different time points was calculated with bivariate regressions. The results are presented in Table 2. and shows that none of the potential confounding variables was significantly associated with one of the CAR values.

**TABLE 2:** Bivariate association between possible confounding variables and BDI-II values or CAR indices.

	*M*	*SD*	*p* value for association with AUCg	*p* value for association with AUCi	*p* value for association with BDI_Intake	*p* value for association with BDI_Discharge	*p* value for association with BDI_6WF	*p* value for association with BDI_6MF
Age (years)	40.00	15.27	0.32	0.54	0.47	0.06	0.04*	0.57
Male	42 (34,4%)		0.58	0.44	0.125	0.032*	0.33	0.47
Female	80 (65.6%)							
Amount secondary diagnosis	2.14	1.51	0.28	0.44	0.004**	0.000***	0.000***	0.000***
Length depressive episode (months)	30.51	92.41	0.57	0.77	0.16	0.000***	0.017*	0.032*
Distance to the first mental illness (years)	18.69	14.47	0.74	0.58	0.26	0.70	0.64	0.11
Medication	106 (86.9%)		0.53	0.09	0.08	0.43	0.44	0.05*
No Medication	8 (6.6%)							
Awakening Time	6.36	0.65	0.12	0.57	0.54	0.38	0.72	0.13

Bivariate association between confounding variables and CAR indices or BDI-II. M, mean; BDI-II, Beck Depressive Inventory II; WF, week follow-up; MF, month follow-up; AUCi, area under the curve increase; AUCg, area under the curve ground. *p < 0.05, **p < 0.01, ***p < 0.001.

### Cortisol awakening response at admission predict follow up depression severity

There was no significant correlation between psychometrics (BDI-II at intake, discharge and 6WF and 6 MF) and CAR values (see Table 3), or between AUCg and age (*r* = 0.115, *p* = 0.23) or AUCi and age (*r* = –0.034, *p* = 0.72).

**TABLE 3:** Correlation between CAR and depressive pathology.

Measure		1. AUCg	2. AUCi	3. CAR + 30	4. CAR + 45	2. BDI-II Intake	3. BDI-II Discharge	4. BDI-II 6WF	5. BDI-II 6MF
1. AUCg	Pearson Correlation								
	Sig. (2-tailed)								
	*N*								
2. AUCi	Pearson Correlation	0.61 (0.71–0.48)							
	Sig. (2-tailed)	<0.001***							
	*N*	117							
3. CAR + 30	Pearson Correlation	0.64 (0.73–0.51)	0.99 (0.99–0.98)						
	Sig. (2-tailed)	<0.001***	<0.001***						
	*N*	117	117						
4. CAR + 45	Pearson Correlation	0.46 (0.59–0.30)	0.90 (0.93–0.86)	0.82 (0.88–0.76)					
	Sig. (2-tailed)	<0.001***	<0.001***	<0.001***					
	*N*	117	117	117					
5. BDI-II Intake	Pearson Correlation	−0.15 (0.03–(−0.32))	−0.02 (0.17–(−0.20))	−0.03 (0.15–(−0.21))	0.03 (0.21–(−0.16))				
	Sig. (2-tailed)	0.11	0.85	0.73	0.78				
	*N*	117	117	117	117				
6. BDI-II Discharge	Pearson Correlation	−0.14 (0.04–(−0.31))	0.06 (0.24–(−0.12)	0.04 (0.22–(−0.14))	0.11 (0.28–(−0.08))	0.46 (0.60–0.32)			
	Sig. (2-tailed)	0.13	0.52	0.67	0.25	<0.001***			
	*N*	117	117	117	117	122			
7. BDI-II 6WF	Pearson Correlation	−0.15 (0.04–(−0.32))	0.11 (0.28–(−0.08)	0.09 (0.27–(−0.09))	0.13 (0.30–(−0.06))	0.53 (0.65–0.39)	0.76 (0.82–0.67)		
	Sig. (2-tailed)	0.12	0.26	0.33	0.17	<0.001***	<0.001***		
	*N*	115	115	115	115	120	120		
8. BDI-II 6MF	Pearson Correlation	−0.21 (0.00–(−0.41))	−0.13 (0.09–(−0.33))	−0.12 (0.10–(−0.33))	−0.12 (0.10–(−0.33))	0.65 (0.76–0.50)	0.61 (0.73–0.45)	0.75 (0.83–0.64)	
	Sig. (2-tailed)	0.05	0.26	0.29	0.27	<0.001***	<0.001***	<0.001***	
	*N*	82	82	82	82	86	86	84	

Two-tailed 95% confidence occur in bracets. M, mean; BDI-II, Beck Depressive Inventory II; WF, week follow-up; MF, month follow-up; AUCi, area under the curve increase; AUCg, area under the curve ground. *p < 0.05, **p < 0.01, ***p < 0.001.

Hierarchical multiple regression analyses with AUCg and AUCi were conducted to predict BDI-II 6MF, after controlling for BDI-II Intake (left column) and BDI-II at Discharge (right column), while controlling for BMI as in our previous study (Eikeseth et al., 2019). BDI-II Intake and BMI were entered in step 1 and the CAR at step 2. All models were significant for the first step where BDI-II intake and BMI were entered. For AUCi, significant results arose only in the model where both BDI-II Discharge (β = 0.753, *p* < 0.01) and AUCi (β = 0.202, *p* > 0.05) were statistically significant individual predictors of BDI-II 6MF (*R*^2^ = 0.578, *F* = 17.82, *p* = 0.026). AUCi in all other regressions was not significant (see Table 4a–d)

**TABLE 4:** Hierarchical regressions.

a. BDI-II Intake*AUCg				b. BDI-II Discharge*AUCg		
	
	BDI-II Discharge	BDI-II 6WF	BDI-II 6MF		BDI-II 6WF	BDI-II 6MF
BDI-II Intake	0.471**	0.532**	0.631**	BDI-II Discharge	0.753**	0.591**
BMI	0.018	0.057	0.045	BMI	0.019	0.030
AUCg	0.067	0.067	0.121	AUCg	0.041	0.148
*F*	11.40	14.99	19.33	*F*	49.76	16.56
*R* ^2^	0.234	0.290	0.430	*R* ^2^	0.576	0.392
Δ*R*^2^	0.004	0.004	0.014	Δ*R*^2^	0.002	0.022

c. BDI-II Intake*AUCi				d. BDI-II Discharge*AUCi		
	
	**BDI-II Discharge**	**BDI-II 6WF**	**BDI-II 6MF**		**BDI-II 6WF**	**BDI-II 6MF**

BDI-II Intake	0.481**	0.543**	0.654**	BDI-II Discharge	0.755**	633**
BMI	0.006	0.040	0.065	BMI	0.009	0.004
AUCi	0.070	0.117	0.145	AUCi	0.063	0.202
*F*	11.43	15.66	19.81	*F*	50.233	17.82*
*R* ^2^	0.234	0.299	0.436	*R* ^2^	0.578	0.410
Δ*R*^2^	0.005	0.013	0.020	Δ*R*^2^	0.004	0.039

BDI, Beck’s Depression Inventory II; BMI, body mass index; AUCg, area under the curve ground; AUCi, area under the curve increase. *p < 0.05; **p < 0.01.

### Comparison with correlations to original study

Eikeseth et al. (2019) found a negative correlation between BDI-II 6WF and CAR *r* = −0.259, *p* = 0.02 and a negative correlation between BDI-II 6MF and CAR *r* = −0.213, *p* = 0.048 (*N* = 79). Compared to the correlations found in this study there was no significant difference in correlation coefficients between these two studies, *z* = 0.065, *p* = 0.474.

The same analytical method (Total cortisol increases after 30 or 45 min: Cortisol 30 or 45 min after awakening minus Cortisol awakening) used by Eikeseth et al. (2019), produced similar results. The total Cortisol increase between awakening and 30 or 45 min after awakening was not significantly correlated with BDI-II 6MF (after 30 min: *r* = −0.12, *p* = 0.29; after 45 min: *r* = −0.12, *p* = 0.27). All CAR values (AUCg, AUCi, total Cortisol increase after 30 min and total Cortisol increase after 45 min) are significantly positively correlated to each other (for all *r* > 0.634, *p* < 0.001).

## Discussion

Using a stricter measurement protocol to examine the value of the CAR in predicting follow-up depressive symptoms in hospitalized patients, the aim of this paper was to replicate our earlier finding that a blunted CAR can predict mood deterioration after an inpatient treatment of severe MDD (Eikeseth et al., 2019). In contrast to Eikeseth et al. (2019) three cortisol samples were used to calculate the CAR. We also used a follow-up measure to check the compliance to the measurement protocol.

Our former reported results (Eikeseth et al., 2019) have been partly replicated. There is a negative association between CAR at the beginning of an inpatient treatment and long term depressive symptoms. Controlling for initial CAR levels, and for symptom levels at 6 weeks and at 6 months, following discharge did not reach statistical significance levels; however, the observed effect size is comparable to our original study.

In this replication study we examined a naturalistic sample of MDD patients with approximately comparable depressive symptoms (BDI-II Intake + 2 BDI-II points compared to the original study), the measurement protocol was stricter and the evaluation methods were more accurate and yet we found a comparable association and comparable effects. The Cortisol-values were a little lower and the cortisol awakening response seems to be weaker than in the original study (see Table 1). Apart from that the clinical naturalistic setting was identical (number of individual therapy units, length of inpatient treatment, amount and type of group therapy, complexity of diagnoses, etc.). The replication of association and effects show that a blunted CAR is associated with a higher depressive symptom deterioration 6-months after discharge of an inpatient treatment (Table 4d). However, the lack of statistical significance in addition to the rather small effect sizes for CAR measurements, suggest only a minor clinical meaningfulness. Other variables, for example depression severity at intake, or at discharge have a higher predictive power for the long term course of MDD symptoms. At the same time, the replicated effect sizes show that the CAR can be seen as a biomarker for depressive severity and predicts the probability for a relapse after discharge.

To the best of our knowledge, this is the first study to investigate the association between CAR and a long-term follow-up after discharge in a naturalistic setting. These findings are in line with previous work indicating that a CAR at the beginning of a therapeutic intervention is associated with a greater treatment outcome after a 4-week inpatient programme (Jones et al., 2015). With only 25 included patients, however, these findings generalizability is rather limited. A higher baseline CAR may predict depressive episode recurrence (Vrshek-Schallhorn et al., 2013). What appears to be contradictory to our findings, may be explained by the dependence of the association between CAR and depression severity and the definition of MDD as categorical or dimensional. Previous research suggested that the association between CAR and depressive symptoms may be described as an inverted U function (Veen et al., 2010; Wardenaar et al., 2011). Patients with mild depressive symptoms show a rather low CAR, which is comparable to healthy control persons. Patients with moderate depressive symptoms show an increased CAR whereas patients suffering from severe depression show a blunted CAR.

Apart from that, a higher and flexible CAR could be associated with successful coping (Dedovic and Ngiam, 2015). For example, resilient psychological profiles (low stress symptoms) appear to be associated with a flexible CAR: low CAR on weekends, higher CAR on weekdays (Schlotz et al., 2004), while vulnerable psychological profiles (high stress) appear to be associated with a rigid CAR (same magnitude during weekdays and weekends). Reduced, inflexible CAR values seem to indicate exhaustion of the stress system. These inflexible profils may explain the difficulties of the HPA axis of depressive patients to adapt to different situations (Dedovic and Ngiam, 2015). Simultaneously high trait rumination in non-depressed individuals seems to be associated with a blunted cortisol reactivity, too (Vrshek-Schallhorn et al., 2019)

Other values seem to have a stronger predictive power for longterm depressive symptoms. For instance, Dedovic and Ngiam (2015) show that a negative attributional style can predict depressive symptoms and they found a negative association between CAR and hopelessness. A family history of depression, moreover, seems to be associated with higher CAR values even if the participants themselves showed no depressive symptoms (Dedovic and Ngiam, 2015).

### Limitations

The findings of this study should be interpreted in light of some limitations. First, Cortisol seems to have a high day to day variation. In addition, adherence to the measurement protocol was not checked with electronic monitoring or a repeated measurement on the next day, there was only a self assessment questionnaire to prove the participant’s “adherence to instruction”. Hellhammer et al. (2007) stated that at least 6 days of measurement are needed to assess the CAR increase and two days for CAR AUC. However, in this study, all patients participated in the same daily clinical routines so that potential confounders such as increased anxiety or anticipation over the day’s activities, are minimized. Nevertheless individual participants’ anxiety or anticipation to these routines may vary and may influence the results, too.

Second, sleep disturbances are one of the most common symptoms of MDD (American Psychiatric Association, 2013), but the subjective self reported assessment of sleep quantity and quality, or the sleep-item in BDI-II is not very meaningful (Tsuchiyama et al., 2003). Additionally, poor sleep can affect the CAR and therefore may have influenced our results (Elder et al., 2014; Santiago et al., 2020). However, since sleep problems are common in depressed patients it can be seen as part of MDD and could not be controlled in a naturalistic setting. Especially because patients often suffer from very heterogeneous sleep problems (sleeping too much, sleeping too little, difficulty falling asleep or staying asleep).

Finally, this study took place in a naturalistic clinical setting and data collection was part of routine diagnostics. Therefore, the control of other possible internal and external influencing factors (such as individual comorbid mental disorders or medication use/dose changes, etc.) was only possible to a limited extent. Findings of MDD and anxiety patients showed either heightened (Greaves-Lord et al., 2007) or lowered CARs (Kallen et al., 2008), therefore it would be interesting to examine differences between the subgroups of these mental disorders in future studies (Kudielka and Wüst, 2010). This is particularly true for naturalistic settings, where more influencing factors compared to laborator studied have to be expected.

### Future directions

To the best of our knowledge this is the first study to investigate the association between CAR and depressive symptom deterioration after an inpatient psychotherapy. Future studies analyzing the predictive power of CAR in MDD patients should consider combining the measurement of CAR with an assessment of sleeping quality and quantity, for example by including actigraph measures during sleep. Sleeping disorders may lead to a disruption in cortisol production which then itself might result in altered cortisol levels after awakening (Dockray et al., 2008; Chida and Steptoe, 2009). The assessment of physical activity could provide a measure for a sedentary lifestyle, which is often found in depressive patients (Zhai et al., 2015) and could have an impact on cortisol levels (Adam et al., 2010).

MDD is a disorder with many different symptom constellations and subtypes that are associated with distinct alteration of the HPA axis (Antonijevic, 2006). Psychophysiological measures are very sensitive to various internal and external influencing factors and the individual interactions of them are not fully understood yet (Mayeux, 2004; Chida and Steptoe, 2009). Therefore it is necessary to use measurement designs and detailed method and result descriptions (Stalder et al., 2016). While a strength of this study is its naturalistic design implemented in a psychosomatic hospital with a patient population resembling a very typical profile for German institutions, future research on more homogeneous samples could differentiate clearer between individual variables contributing to the observed effect. Sub-samples of MDD patients with blunted or increased CAR could be further differentiated. Future studies could also focus on a more precise symptom dependent sample formation (i.e., subgroups of depression with main symptoms like rumination, hopelessness, anxiety, exhausting, restlessness, depressive severity) instead of sample formation depending on diagnosis only.

## Conclusion

This replication resulted in statistically non-significant moderate predictive power of CAR on depressive severity 6-weeks and 6-months after discharge, thus largely replicating previous (significant) findings. Improved assessment and monitoring protocols add to the robustness of these findings. Therefore, the CAR at intake appears to be a (limited) predictive biomarker for global depressive symptoms after treatment and discharge. A blunted CAR at the beginning of a psychotherapy seems to be not statistically significant associated with depressive symptom severity 6 months after discharge and should therefore not be further regarded following the logic of significance testing with pre-determined p-values. Nevertheless it can be stated that the effect size is very similar to the previous study, adding to the impression of robust results.

## Data availability statement

The raw data supporting the conclusions of this article will be made available by the authors, without undue reservation.

## Ethics statement

This study was approved by the Ethics committee of the “Medical Association Westfalen-Lippe” and written informed consent was obtained from all participants prior to data collection. The patients/participants provided their written informed consent to participate in this study.

## Author contributions

SN, MW, and SS contributed to the conception and design of the study. SN and MP organized the database. SN, SS, MC, and RL performed the statistical analysis. SN wrote the first draft of the manuscript. All authors contributed to the manuscript revision, read, and approved the submitted version.

## Footnotes

1 All tests were justified conservatively using a two-tailed test, although we could assume a directed hypothesis.
